# Patient-Reported Outcome Measures in the Elderly: Do These Reflect Healing Post-Fragility Fracture of the Pelvis?

**DOI:** 10.7759/cureus.92727

**Published:** 2025-09-19

**Authors:** Samantha E Bartman, Cari Whyne, David Stephen, Diane Nam

**Affiliations:** 1 Institute of Biomedical Engineering, University of Toronto, Toronto, CAN; 2 Orthopaedic Biomechanics Lab, Sunnybrook Research Institute, Toronto, CAN; 3 Division of Orthopaedics, Department of Surgery, University of Toronto, Toronto, CAN; 4 Division of Orthopaedics, Sunnybrook Health Sciences Centre, Toronto, CAN

**Keywords:** elderly, fragility fractures of the pelvis, healing, non-operative management, patient reported outcome measures

## Abstract

Background and objective

Fragility fractures of the pelvis (FFPs) have become increasingly common in the geriatric population. The prolonged healing process associated with current non-operative management of FFPs has a significant negative impact on patient mobility, independence, and quality of life (QoL). This study aimed to document functional outcomes and QoL measures in individuals with non-operatively treated FFPs and examine if a relationship exists between these parameters and healing status.

Methods

This was a prospective case series involving a cohort from a single level 1 trauma center. Fifty-three elderly patients (age ≥65 years) who, after a fall from less than 5 feet, sustained a non-operative FFP as diagnosed on X-ray between 2008 and 2019. Functional outcomes using Musculoskeletal Function Assessment (MFA) and QoL measures based on 36-Item Short Form Health Survey (SF-36) were collected over two years. Healing was assessed per available follow-up X-ray imaging by four orthopaedic surgeons.

Results

Health status and function did not improve in elderly patients with stable FFPs treated non-operatively from reported baseline levels, with many MFA and SF-36 categories demonstrating a steady decline out to approximately 24 months. Follow-up X-ray imaging was only available for a subset of these individuals (n = 35). Substantial agreement was found between all four surgeon raters (Fleiss Kappa = 0.65) with respect to their evaluation of healing status. Confirmation of bone healing on X-ray (level 3) was found for only seven individuals. There was no association found between any MFA or SF-36 category and healing status for patients with coincident reporting (n = 16).

Conclusions

Overall, patient function and QoL decrease post-stable FFP, with no relationship evident between these measures and healing status. Worsening patient-reported outcome measures (PROMs) regardless of healing status should prompt discussions and further investigations regarding the need for continued monitoring of non-operative FFPs post-injury.

## Introduction

The prevalence of fragility fractures is projected to rise significantly as the elderly population grows globally, intensifying the already considerable burden these injuries place on the healthcare system [1-4]. Currently, the direct healthcare costs associated with fragility fracture management in Canada amount to 1.1 billion dollars annually [1]. Beyond the significant economic impact, fragility fracture represents a significant challenge due to the profound negative effect they have on patients, as reflected by the high mortality rates post-fracture [2,3,5-7]. Often resulting from a low-energy injury, such as a fall from standing height, fragility fractures are indicative of underlying conditions that negatively impact bone quality and strength (e.g., osteoporosis), increasing fracture susceptibility [3,7-9]. These fractures predominantly occur at the wrist, shoulder, spine, hip, and pelvis, with the spine and hip generally being the most vulnerable to fracture [3,8]. In recent years, the incidence of fragility fractures of the pelvis (FFPs) has risen. In the majority of FFPs, the posterior pelvic ring remains intact and, as such, is considered biomechanically stable [8,9].

Stable pelvic fractures, including incomplete sacral fractures, unilateral rami fractures, and bilateral rami fractures, are unlikely to displace [8,10]. Hence, the management of these fractures is primarily non-operative, emphasizing effective pain control to alleviate discomfort and early mobilization to promote functional recovery [7-9,11]. Although stable pelvic fractures may heal, pain and impaired physical function often persist [5,6,9,11-14]. The prolonged healing process often associated with current non-operative FFP management has a significant negative impact on patient mobility, independence, and quality of life (QoL) [5,6,9,11-13]. The vulnerability of the geriatric population is further reflected in the considerable mortality rates at one year (16%) and five years (30-58%) following a stable FFP [6,11-13].

Patient-reported outcome measures (PROMs) such as the 36-Item Short Form Health Survey (SF-36) and Musculoskeletal Function Assessment (MFA) have been used to understand the impact of fragility fractures on function and QoL. Fragility fractures of the hip are highly prevalent and have been reported to yield a significant negative impact on patient function and QoL, as well as high mortality rates post-fracture (14-58% one year post-fracture)[15]. Unlike FFPs, most hip fractures are treated operatively, followed by adequate pain control and physical therapy [15]. PROMs have also been used to assess clinical outcomes of current operative hip fracture management and have provided insights into the decline of mobility, independence, and QoL post hip fracture in this patient population [16,17]. While the SF-36 has demonstrated strong responsiveness to clinical change in elderly patients with hip fragility fractures, the MFA or its shorter version, the SMFA, has not been extensively studied in these populations [18-20]. Nevertheless, the MFA has proven capable of detecting clinical change across a variety of musculoskeletal conditions, most notably following trauma [19,21].

Recent studies have used PROMs to highlight the significant negative impact predominantly operatively treated FFPs have on patients, with many reporting increased pain, decreased mobility, and loss of ability to perform many activities of daily living (ADL) independently [11,12,22-24]. However, literature focusing on functional outcomes and QoL measures following solely non-operatively treated FFPs is lacking [25,26]. The relationship between radiographic healing and PROMs remains unclear [27-30]. In distal radius fractures and unstable pelvic ring injuries, radiographic alignment has shown poor or no correlation with function or QoL, whereas in hip fractures, optimal reduction has been correlated with improved function [27-30]. It has not been established whether a relationship exists between clinical healing, physical function, and QoL following a non-operatively treated FFP. An understanding of the impact of current stable FFP management is needed to establish how this patient population may compare to normative populations and those with other more common injury patterns (i.e., hip fractures). It is also important to determine if there is a correlation between the healing of FFP and PROMs. Hence, we performed this study to document functional outcomes and QoL measures in individuals with FFPs treated non-operatively and examine if a relationship exists between these parameters and imaging-based healing status.

## Materials and methods

Participants

This was a retrospective analysis of a limited cohort prospectively collected from a single institution’s pelvic and acetabular database (REB PIN 5528). This database contains demographics, diagnosis, treatment, and PROMs for individuals treated for a pelvic or acetabular fracture at a level 1 trauma center, between 1998 and 2021. While this database focuses on clinical outcomes associated with surgical treatment of pelvic and acetabular fractures following high-energy trauma, it also encompasses a unique subset of cases between 2008 and 2019. This subset comprises elderly patients (aged ≥65 years) who, after a fall from less than 5 feet, sustained a non-operative FFP as diagnosed on X-ray.

Quality of life and functional outcomes

For this study, SF-36 and MFA were used to assess QoL and functional outcomes. The SF-36 consists of 36 questions classified into eight categories: physical functioning, role limitations due to physical problems, role limitations due to personal or emotional problems, bodily pain, general health, general mental health, social functioning, and vitality [31]. The MFA consists of 100 questions divided into 10 categories: mobility, fine motor, housework, ADL, sleep, leisure, relationships, cognition, emotional adjustment, and employment [19]. A higher SF-36 score and a lower MFA score indicate better health status [19,31]. The SF-36 and MFA scores were calculated for each patient at each of the time points where they completed the survey (at injury, six months post-injury, 12 months post-injury, and 24 months post-injury). Patients with missing surveys at a given time point were excluded from analysis for that time point but remained included in the overall cohort. Missing data were not estimated.

Timeline until healing

Two senior orthopaedic trauma surgeons and two senior orthopaedic surgery residents assessed each set of patient X-rays and provided a score indicating the degree to which the fracture is healed (1 = less than 50% healed, 2 = 50-75% healed, 3 = above 75% healed). A score of 1 or 2 constituted an unhealed fracture, and a score of 3 constituted a healed fracture. The number of patients deemed healed within six months of injury was quantified. It should be noted that a score of 1 was automatically provided to X-rays taken at the time of injury. Inter-rater variability analysis was used to determine the agreement between the scores provided by the senior orthopaedic surgeons and orthopaedic surgical residents.

Statistical analysis

The study population was characterized using descriptive statistics, including mean, median, standard deviation (SD), and interquartile range (IQR). PROMs were collected longitudinally at injury, six months, 12 months, and 24 months post-injury, and were summarized at each time point to illustrate general trends in physical function and QoL over the two-year follow-up period. No statistical comparisons were performed between time points. To contextualize patient outcomes, unpaired two-sample t-tests were conducted to compare PROMs from this cohort of patients with non-operatively managed stable FFPs at each of the four follow-up time points with published normative values. Normative values for the SF-36 were determined using a population consisting of individuals aged ≥75 years, and normative values for the MFA were determined using a population where the majority (60.6%) of the population were aged ≥56 years [32, 33]. SF-36 scores at six and 12 months post-FFP were also compared to scores reported in the literature secondary to fragility fractures of the hip. P-values <0.05 were considered statistically significant.

## Results

**Figure 1:**
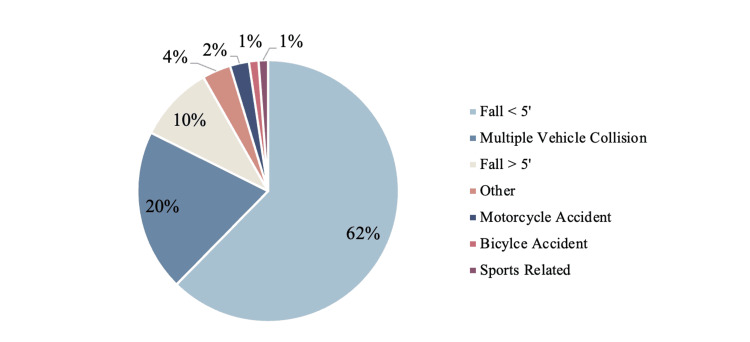
Pelvic fractures in the elderly by mechanism of injury (sorted by prevalence)

Between 2008 and 2019, this database recorded the data of 107 elderly patients who were diagnosed with a pelvic fracture at a single level 1 trauma center. Of these, 85 were treated non-operatively with adequate pain control and early mobilization. These patients were further categorized by the mechanism of injury; 17 (20%) were a result of a multiple vehicle collision (seven (8%) were occupants and 10 (12%) were pedestrians), two (2%) were a result of a motorcycle incident, one (1%) was a result of a bicycle accident, eight (9%) were a result of a fall from greater than 5 feet, 53 (62%) were a result of a fall from less than 5 feet, one (1%) was a result of a sports-related accident, and three (4%) were a result of other mechanisms (Figure 1). 

Follow-up data were somewhat limited for the subset of 53 patients who sustained a non-operative FFP following low-energy injury. The baseline characteristics of the study population can be found in Table 1. Follow-up X-rays were available for 35 patients, and follow-up survey data were available for 40 patients. Scores on the SF-36 and MFA at injury, six months post-injury, 12 months post-injury, and 24 months post-injury are presented in Table 2. 

**Table 1:** Baseline characteristics of the cohort BMI: body mass index; SD: standard deviation

Characteristic		Values (N = 53)
Age at the time of injury, years, mean ± SD {range}		81.04 ± 8.32 {67-97}
Sex, n (%)		
	Female	45 (84.9%)
	Male	8 (15.1%)
Injury Severity Score (ISS), median (IQR) {range}		4 (0) {1-21}
BMI, kg/m^2^, mean ± SD {range}		23.46 ± 3.64 {16.79-32.0}
Race, n (%)		
	White/Caucasian	48 (90.6%)
	Asian or Pacific Islander	1 (1.9%)
	Hispanic	1 (1.9%)
	First Nations or Native American	1 (1.9%)
	Other	1 (1.9%)
	Unknown	1 (1.9%)
Comorbidities per patient, median (IQR) {range}		3.82 (3) {0-11}
Comorbidity prevalence, n (%)		
	Arthritis	28 (52.8%)
	Back problems	24 (45.3%)
	Hearing problems	17 (32.1%)
	Heart problems	15 (28.3%)
	Blood pressure	14 (26.4%)
	Stomach problems	11 (20.8%)
	Cancer	9 (17%)
	Depression	9 (17%)
	Stroke	6 (11.3%)
	Vision problems	6 (11.3%)
	Diabetes	5 (9.4%)
	Lung problems	5 (9.4%)
Education, n (%)		
	Less than high school	8 (15.1%)
	Graduated from high school	8 (15.1%)
	Some college/university	9 (17%)
	Graduated from college/university	17 (32.1%)
	Postgraduate school or degree	10 (18.9%)
	Unknown	1 (1.9%)
Smoking, n (%)		
	Smoke 1-10 cigarettes a day	4 (7.5%)
	Smoke pipe or other	2 (3.8%)
	Former smoker	17 (32.1%)
	Never	30 (56.6%)

**Table 2:** Average SF-36 and MFA scores (± SD) at each time point Higher SF-36 and lower MFA scores indicate better health status SD: standard deviation; SF-36: 36-Item Short Form Health Survey; MFA: Musculoskeletal Function Assessment

	0 months post-injury (N = 53)	6 months post-injury (N = 40)	12 months post-injury (N = 39)	24 months post-injury (N = 39)
SF-36				
Physical Functioning	60.0 ± 37.7	45.0 ± 33.3	44.3 ± 31.6	46.0 ± 33.5
Role Limitations Due to Physical Problems	58.5 ± 40.7	51.9 ± 44.7	60.3 ± 41.3	46.8 ± 41.4
Role Limitations Due to Emotional Problems	76.1 ± 36.6	65.0 ± 42.0	67.5 ± 42.2	69.2 ± 39.3
Vitality	66.3 ± 20.6	52.8 ± 22.5	51.8 ± 21.2	51.9 ± 21.6
Mental Health	80.2 ± 8.8	74.3 ± 18.5	73.9 ± 18.7	72.1 ± 22.0
Social Functioning	81.1 ± 22.8	80.3 ± 23.3	76.0 ± 28.9	76.6 ± 22.4
Pain	75.7 ± 23.3	65.9 ± 27.4	66.0 ± 24.3	66.0 ± 25.7
General Health	70.6 ± 20.9	61.8 ± 21.7	58.3 ± 22.1	62.6 ± 20.1
MFA				
Mobility	54.0 ± 16.6	61.5 ± 17.8	60.5 ± 17.9	61.2 ± 20.3
Fine Motor	29.7 ± 20.7	20.0 ± 25.4	20.9 ± 27.4	21.2 ± 25.8
Housework	28.5 ± 29.9	47.8 ± 30.8	49.6 ± 31.3	50.1 ± 33.4
Activities of Daily Living	14.4 ± 19.3	21.5 ± 23.9	24.9 ± 27.1	23.8 ± 22.5
Sleep	30.2 ± 24.9	33.3 ± 28.2	31.6 ± 28.3	34.2 ± 31.1
Leisure	30.7 ± 36.9	46.3 ± 37.8	50.6 ± 38.7	46.8 ± 39.4
Relationships	12.1 ± 15.5	14.3 ± 20.1	19.7 ± 23.3	17.4 ± 20.7
Cognition	19.3 ± 29.3	29.4 ± 35.8	32.7 ± 39.0	39.7 ± 37.5
Emotional Adjustment	24.5 ± 22.7	31.4 ± 26.4	32.2 ± 28.5	33.5 ± 28.4
Employment	7.5 ± 26.7	7.5 ± 26.7	2.6 ± 16.0	7.7 ± 27.0

Overall, patient SF-36 scores decreased from baseline, although there was some variability in Role Limitation due to Physical Problems category. Conversely, patient MFA scores generally increased from baseline, excluding the Fine Motor and Employment categories. This trend in SF-36 and MFA scores indicates that as time passes, function and QoL typically worsen in these individuals. Generally, SF-36 and MFA scores stabilized after six months post-injury. 

To further assess the impact of FFPs, SF-36 and MFA scores at six months, 12 months, and 24 months post-injury were compared to normative data from the general population (Table 3). Patients with FFPs reported significantly lower SF-36 scores in categories such as Physical Functioning, Role Limitations Due to Emotional Problems, Vitality, Mental Health, and General Health at the six-month, 12-month, and 24-month post-injury time points. Patients also reported significantly lower SF-36 scores in the Role Limitations Due to Physical Problems category at the 24-month post-injury time point. With respect to MFA, FFP patients reported significantly higher scores in the Mobility, Housework, and Activities of Daily Living categories at the 6-month, 12-month, and 24-month post-injury timepoints compared to normative data. In contrast, significantly lower scores in the Leisure and Employment categories at 12 months post-injury and in the Cognition category at 24 months post-injury were reported by those with FFPs compared to normative data.

**Table 3:** Comparison of SF-36 and MFA scores post-FFP to the general population at each time point Normative values for the SF-36 were determined using data from Hopman et al., using a population consisting of individuals aged ≥75 years [32]. Normative values for the MFA were determined by data from McMichael et al., using a population where the majority (60.6%) of the population were aged ≥56 years [33]. Change is reported as a percent difference with respect to the normative values. Unpaired two-sample t-tests were used to establish if significant differences existed between groups. Bolded, italicized values imply statistical significance SF-36: 36-Item Short Form Health Survey; MFA: Musculoskeletal Function Assessment; FFP: fragility fractures of the pelvis

	0 months post-FFP vs. the general population			6 months post-FFP vs. the general population			12 months post-FFP vs. the general population			24 months post-FFP vs. the general population		
	Percent difference	T-value	P-value	Percent difference	T-value	P-value	Percent Difference	T-value	P-value	Percent Difference	T-value	P-value
SF-36												
Physical Functioning	-1.52	0.23	0.817	23.86	3.20	0.001	25.09	3.32	<0.001	22.22	2.93	0.003
Role Limitations Due to Physical Problems	6.55	0.70	0.483	17.13	1.59	0.111	3.74	0.34	0.735	25.25	2.33	0.020
Role Limitations Due to Emotional Problems	5.23	0.88	0.382	19.05	2.77	0.006	15.91	2.29	0.022	13.78	1.99	0.047
Vitality	-8.51	1.90	0.058	13.67	2.64	0.009	15.16	2.92	0.004	15.02	2.89	0.004
Mental Health	-1.01	0.38	0.707	6.42	2.10	0.036	6.99	2.27	0.023	9.16	2.95	0.003
Social Functioning	2.52	0.67	0.504	3.47	0.80	0.421	8.70	1.96	0.050	7.93	1.81	0.070
Pain	-8.45	1.69	0.092	5.53	0.97	0.333	5.41	0.93	0.350	5.41	0.93	0.351
General Health	0.84	0.24	0.811	13.27	3.26	0.001	18.07	4.42	<0.001	12.04	2.96	0.003
MFA												
Mobility	-89.74	8.39	<0.001	-116.09	9.48	<0.001	-112.62	9.08	<0.001	-114.88	9.19	<0.001
Fine Motor	-115.37	4.52	<0.001	-45.04	1.52	0.129	-51.41	1.71	0.088	-54.07	1.79	0.074
Housework	-19.55	1.27	0.205	-100.41	5.73	<0.001	-107.94	6.07	<0.001	-110.33	6.14	<0.001
Activities of Daily Living	-81.13	2.46	0.014	-170.79	4.47	<0.001	-213.57	5.42	<0.001	-199.24	5.22	<0.001
Sleep	19.08	1.54	0.124	10.68	0.76	0.451	15.26	1.06	0.288	8.39	0.57	0.566
Leisure	13.59	0.94	0.350	-30.17	1.85	0.065	-42.53	2.56	0.011	-31.71	1.91	0.057
Relationships	35.02	1.91	0.057	23.47	1.09	0.276	-6.03	0.27	0.785	6.36	0.30	0.764
Cognition	21.26	1.08	0.282	-19.85	0.88	0.381	-33.38	1.44	0.151	-62.15	2.68	0.008
Emotional Adjustment	6.27	0.47	0.641	-20.08	1.34	0.181	-23.16	1.51	0.131	-28.06	1.84	0.067
Employment	31.94	0.94	0.349	31.94	0.83	0.408	76.73	2.03	0.043	30.20	0.77	0.441

Patient SF-36 scores at six months and 12 months following a stable FFP were also compared to SF-36 scores at identical time points following a surgically treated hip fracture (Table 4) [16,17]. (Note: MFA scores after sustaining an operatively treated hip fracture were not available for comparison.) Elderly patients who sustained a non-operative pelvic fracture after a low-energy fall reported significantly worse SF-36 scores in the Role Limitations Due to Emotional Problems, Vitality, and Pain categories and significantly better SF-36 scores in the Role Limitations Due to Physical Problems and Social Functioning categories six months post-injury than those with hip fractures. However, no significant differences were found between those who sustained a non-operative pelvic fracture and those who sustained an operative hip fracture at the 12-month post-injury time point.

**Table 4:** Comparison of SF-36 scores post-FFP to those post hip fracture at 6- and 12-months timepoints SF-36 scores post hip fracture were sourced from studies by Su et al. and Peterson et al. examining PROMs in the elderly (patients aged ≥65 years) following a hip fracture [16,17]. Change is reported as a percent difference with respect to the established post-hip fracture values. Unpaired two-sample t-tests were used to establish if significant differences existed between groups. Bolded, italicized values imply statistical significance SF-36: 36-Item Short Form Health Survey; FFP: fragility fractures of the pelvis; PROMS: patient-reported outcome measures

	6 months post-FFP vs. 6 months post-hip fracture			12 months post-FFP vs. 12 months post-hip fracture		
	Percent difference	T-value	P-value	Percent difference	T-value	P-value
SF-36						
Physical Functioning	-11.66	0.91	0.3655	3.75	0.23	0.818
Role Limitations Due to Physical Problems	-193.24	5.20	<0.001	-2.13	0.14	0.891
Role Limitations Due to Emotional Problems	22.23	2.76	0.007	7.50	0.59	0.559
Vitality	13.58	2.01	0.047	7.43	0.83	0.407
Mental Health	-6.91	1.43	0.154	0.21	0.04	0.966
Social Functioning	-23.27	2.95	0.004	-1.28	0.14	0.888
Pain	15.90	2.74	0.007	4.31	0.47	0.637
General Health	-2.37	0.38	0.708	14.22	1.85	0.069

Scores indicating the degree of healing of the FFPs on X-ray imaging were compared between two senior orthopaedic trauma surgeons and two orthopaedic surgery residents (PGY3). Based on the inter-rater variability analysis, Cohen’s kappa indicated moderate agreement (kappa = 0.58) between the two senior surgeons and substantial agreement (kappa = 0.74) between the two surgical residents. Fleiss' kappa was also calculated to determine the agreement between all four raters and found to be 0.65, indicating substantial agreement. Overall, the difference between scores provided by the senior surgeons and surgical residents was not significant. 

Of the 53 patients, only 35 (66%) underwent follow-up X-ray imaging (Table 5). The healing scores provided by all four raters were averaged, allowing for each patient to be categorized as healed (a score of 3) or not healed (a score of less than 3). The number of X-rays declined rapidly after three months post-fracture, despite limited healing seen at this time point. Of the patients with X-ray imaging, six were deemed to be healed within six months of injury (three were healed at three months, and an additional three were healed at six months). Confirmation of bone healing on X-ray was found for only seven patients within 12 months of injury. 

**Table 5:** Temporal acquisition of X-ray imaging and assessment of healing in stable FFPs Additional follow-up X-rays were not expected or available on those deemed healed at earlier time points FFP: fragility fractures of the pelvis

	0 months post-FFP	3 months post-FFP	6 months post-FFP	9 months post-FFP	12 months post-FFP
Number of X-rays	53	35	9	5	2
Number of patients deemed healed	0	3	3	1	0

**Figure 2:**
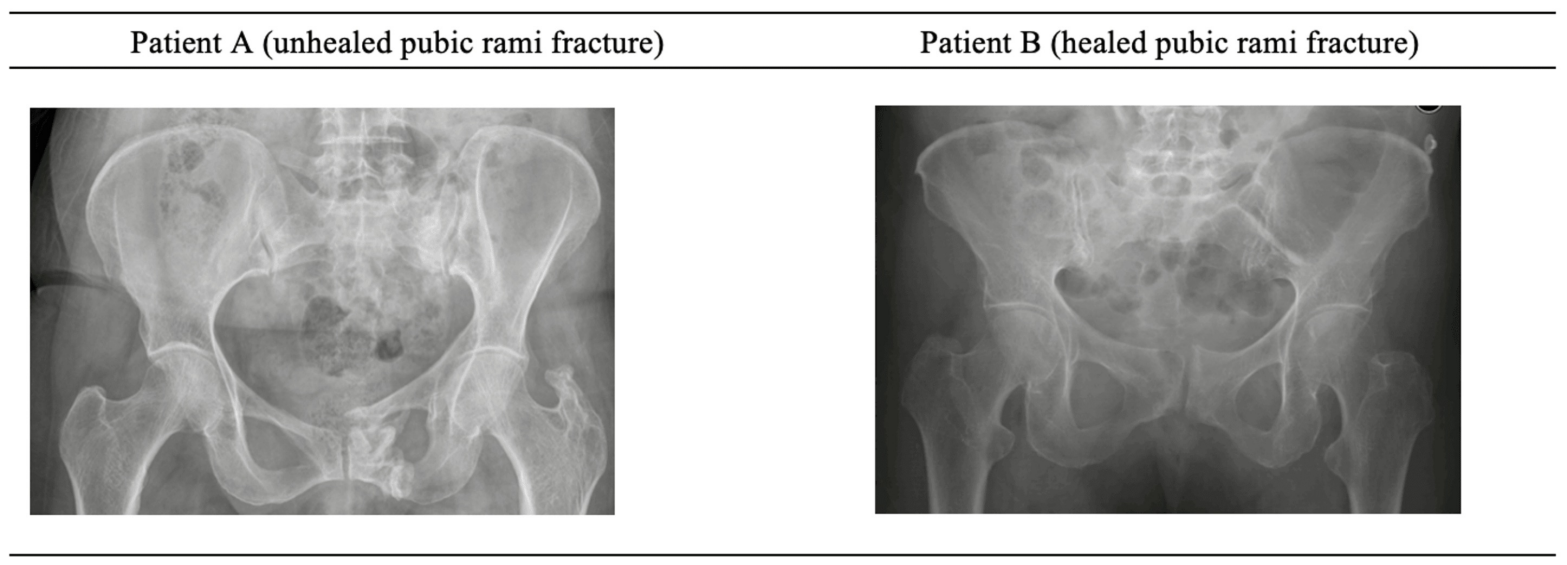
X-ray imaging at final follow-up for two patients in this cohort Patient A with an unhealed pubic rami fracture and Patient B with a healed pubic rami fracture

Due to a lack of temporally paired data points, it was not possible to detect a relationship between healing status and functional outcomes and QoL measures. In qualitatively examining the data, it was clear that there were unhealed patients (e.g., Patient A) who had high SF-36 scores and low MFA scores, indicating good health status, and there were healed patients (e.g., Patient B) who had low SF-36 scores and high MFA scores, indicating poor health status. Figure 2 illustrates the X-ray imaging of Patient A and Patient B obtained at final follow-up, and Table 6 summarizes the SF-36 and MFA scores for each patient recorded at the time these radiographs were acquired.

**Table 6:** Discrepancy between bone healing status and PROMs in patients with stable FFPs Good health status is seen for Patient A based on high SF-36 scores and low MFA scores despite a lack of radiographic evidence of fracture healing. In contrast, Patient B appears healed on X-ray but had low SF-36 scores and high MFA scores, indicating poor health status PROMS: patient-reported outcome measures; FFP: fragility fractures of the pelvis; SF-36: 36-Item Short Form Health Survey; MFA: Musculoskeletal Function Assessment

	Patient A (unhealed pubic rami fracture)	Patient B (healed pubic rami fracture)
SF-36		
Physical Functioning	70.0	0
Role Limitations Due to Physical Problems	100	0
Role Limitations Due to Emotional Problems	100	0
Vitality	95.0	45.0
Mental Health	92.0	52.0
Social Functioning	100	37.5
Pain	77.5	32.5
General Health	75.0	50.0
MFA		
Mobility	45.0	90.0
Fine Motor	14.3	71.4
Housework	0	88.9
Activities of Daily Living	0	33.3
Sleep	0	50.0
Leisure	0	100
Relationships	0	50.0
Cognition	0	75.0
Emotional Adjustment	0	77.8
Employment	0	0

## Discussion

This study focused on assessing the clinical outcomes of elderly patients who, after a fall from less than 5 feet, sustained a non-operative FFP as diagnosed on X-ray. The mean age at the time of injury was 81.04 ± 8.32 years (range: 67-97 years), and the patient cohort was predominantly female (n = 45, 84.9%) and Caucasian (n = 48, 90.6%). This demographic distribution aligns with the well-established relationship between postmenopausal bone loss and fracture risk, with the decline in estrogen following menopause leading to decreased bone density, significantly elevating the risk of sustaining a fragility fracture [2,7,34,35]. Cauley et al. found that Caucasian females have the highest fragility fracture rates among ethnic groups, which may be attributed to lower bone mineral density [34].

Examining the SF-36 and MFA scores of this patient cohort at various time points revealed that functional outcomes and QoL measures did not improve from baseline, with many categories demonstrating a decline out to approximately 24 months. This suggests that non-operatively managed stable FFPs have a lasting impact on mobility, independence, and QoL. When compared to the age-matched general population, this cohort was found to have significantly worsened physical function, vitality, mental health, and general health. The absence of improvement in physical function and mobility contrasts with the expected recovery of movement after fracture healing, underscoring the broader decline observed in this population. The lack of healing in this cohort is further exemplified by the fact that confirmation of bone healing on X-ray was found for only seven of the 53 patients within 12 months of injury.

While literature has primarily focused on the adverse effects associated with surgical management of unstable FFPs, studies examining clinical outcomes of both non-operatively and operatively treated FFPs are scarce [25,26]. A retrospective study by Rommens et al. examined 138 elderly patients (mean age: 80.6 years; 84.8% female) with type 1 FFPs (unilateral or bilateral pubic ramus fractures) and found that patients experienced reduced mobility and independence, with the number of patients living at home dropping from 80.5 to 65.3% [11]. The profound impact of FFPs was also highlighted by the threefold increase in one-year mortality rate (19.1%) compared to the age-matched general population (5.9% for males and 4% for females) [11]. While investigating the long-term outcomes of 187 elderly patients (mean age: 79 years; 32% female) with FFPs, Banierink et al. reported similar findings [12]. Although this cohort included patients with all FFP types, the majority were classified as either type 1 (60%) or type 2 (27%) FFPs, with 98% of patients being treated non-operatively [12]. At roughly two years post-fracture, patients showed a decline in function and overall health status, as measured by PROMs (SMFA and EQ-5D), compared to the age-matched general population [12]. Notably, neither this study nor the work by Rommens et al. assessed fracture healing status.

When compared to elderly patients who sustained an operatively treated hip fracture, this cohort reported heightened pain and a decline in vitality at six months post-fracture. No significant differences were found between those who sustained a non-operative FFP and those who sustained an operative hip fracture at 12 months post-fracture. These findings further highlight the substantial negative impact that current non-operative management of stable FFPs can have on physical function and QoL. The similarities between the clinical outcomes of operatively treated hip fractures and non-operatively treated FFPs further challenge the notion that FFPs are less severe fractures [5,36].

Despite the significant differences in physical function and health status between this cohort and the general population, the presence or absence of bone healing does not appear to correlate with PROMs. This suggests that unhealed FFPs may not negatively impact functional outcomes or QoL measures. Similarly, other comorbidities common in this cohort may have a more significant influence on function and health status than the confirmed presence of bone healing. It is important to acknowledge that this study is limited by the lack of baseline SF-36 and MFA scores, which prevents a conclusion from being made regarding whether diminished function and QoL are a result of current non-operative management of FFPs or pre-existing comorbidities.

The inability to detect a relationship between healing status and PROMs may be attributed to the small number of temporally paired data points (n = 16 with both X-ray and PROMs). As this database primarily focuses on clinical outcomes of surgical treatment of pelvic and acetabular fractures following high-energy trauma, the number of patients that met the inclusion criteria for this analysis was relatively limited. Lack of follow-up pelvic X-ray imaging, even without documented healing, may be due to the absence of established treatments that address the lack of bone union, limiting clinical requests for follow-up imaging. Poor health status and access issues may also have prevented some patients from returning to the clinic for in-person follow-up and X-ray imaging. A more extensive dataset would be required to establish a clear correlation between healing status, function, and QoL.

Despite the small number of follow-up X-rays, this patient cohort highlights the limited healing that may occur in non-operatively treated FFPs. Fibrous non-union may provide sufficient stability in these low-demand patients, allowing some individuals without fracture union to report high SF-36 and low MFA scores. The absence of a validated tool for assessing bias in radiographic-based fracture healing classification is a further limitation. Although this was addressed through the use of clear criteria and multiple independent raters, with inter-rater analysis demonstrating good agreement, the development of validated bias-assessment tools would enhance reliability. Moreover, the lack of specific outcome measures directly related to function post pelvic injury, which is not necessarily reflected in these more generalized PROMs, may contribute to the lack of a clear association.

## Conclusions

Given the rise in aging populations worldwide, the economic and healthcare burden associated with fragility fractures is projected to only increase. This analysis demonstrates that patient function and QoL generally worsen over the 24 months following a stable FFP, regardless of the status of bone healing. Current clinical practice may reflect this understanding, as evidenced by the rapid decline in follow-up imaging past three months post-injury in patients with stable FFPs. The findings of this study should prompt further discussions regarding the suitability of current PROMs to accurately reflect the disability attributed to FFPs as well as the value of continued monitoring of non-operatively managed FFPs post-injury.
